# Design, Testing, and Experimental Validation of a Rotary Vibration-Assisted Polishing Device (RVAPD) for Enhanced Machining and Surface Quality

**DOI:** 10.3390/mi15101242

**Published:** 2024-10-09

**Authors:** Silin Liu, Yan Gu, Jieqiong Lin, Zisu Xu, Tianyu Gao, Xinyang Liu, Xiaoming Zhang, Bingjin Yu

**Affiliations:** 1 Jilin Provincial Key Laboratory of Micro-Nano and Ultra-Precision Manufacturing, School of Mechatronic Engineering, Changchun University of Technology, Yan’an Ave 2055, Changchun 130012, China; 13756896298@163.com (S.L.); linjieqiong@ccut.edu.cn (J.L.); 15568607008@163.com (Z.X.); 15981025677@163.com (T.G.); 13944023947@163.com (X.L.); zxm20011019@163.com (X.Z.); yubingjinccut@163.com (B.Y.); 2 Jilin Provincial Key Laboratory of International Science and Technology Cooperation for High Performance Manufacturing and Testing, School of Mechatronic Engineering, Changchun University of Technology, Yan’an Ave 2055, Changchun 130012, China

**Keywords:** compliant mechanism, vibration-assisted polishing, compliance matrix method, finite element analysis, flexure design

## Abstract

A rotary vibration-assisted polishing device (RVAPD) is designed to enhance polishing force by converting PZT’s linear motion into the rotary motion of a central platform via a flexible mechanism, improving material surface quality. The RVAPD is optimized, simulated, and tested to meet high-frequency and large-amplitude non-resonant vibration polishing requirements. Its structure, designed using theoretical models and finite element software, offers a wide range of polishing parameters. Performance parameters are validated through open-loop tests, confirming effectiveness in polishing experiments. The lever mechanism and Hoeckens connection enhance vibration parameters and motion efficiency, reducing surface flaws in SiC and improving uniformity. Adjusting the RVAPD structure and using the proposed method significantly improve SiC surface quality.

## 1. Introduction

Silicon carbide (SiC) ceramics have a high breakdown voltage and stiffness ratio [1,2,3], and a low thermal coefficient, which makes them widely used in high-temperature semiconductor devices, optical components, and aerospace structures [4]. Due to prolonged exposure to high temperatures in oxygen-enriched environments, SiC is prone to oxidation [5]. The SiO2 layer that forms plays a protective role, but its volume expansion and internal stress cause crack formation, thereby reducing the performance of thermal components in aircraft engines [6] and the thermal protection systems of high-speed vehicles [7,8]. During SiC processing, surface defects such as microscopic cracks and pits typically appear randomly on the material’s surface [9]. Poor surface quality accelerates oxidation and corrosion rates [10,11] and affects the frictional properties and other parameters [12]. Therefore, surface treatment of SiC ceramics is necessary. Vibration-assisted polishing, as a precision machining technology, is effective in removing surface roughness peaks on SiC ceramics, improving surface morphology and enhancing material performance [13,14].

The introduction of vibration has enhanced polishing by increasing material removal rate, reducing machining time, and minimizing damage [15,16,17,18]. Non-resonant vibration modes have been effectively applied for high-precision and complex shapes [19]. By optimizing vibration parameters, machining performance, material removal, and surface quality have improved [20,21,22]. The non-resonant vibration-assisted polishing device adjusts vibration parameters and processing trajectory for high-precision polishing [23]. Vibration frequency and amplitude interactions influence performance; balancing them is crucial. Effective transmission is closely related to trajectory vibration, and the complexity of the connection mechanism can hinder cross-coupling movement. High-frequency operation requires precise design and control to mitigate adverse effects. Using notched flexure hinges improves efficiency and compliance [24,25], and optimizing hinge size and arrangement can enhance performance and extend service life [26]. Gu et al. designed a polishing device with a low frequency of 237.51 Hz and a high stroke of 83 μm using a bridge structure and lever mechanism. They also introduced a magnetorheological process that improves surface quality by increasing tangential force. However, the transmission chain mechanism increases mass, limiting performance and resulting in a vibration amplitude of 26 μm at 706.54 Hz [27,28]. Gu et al. further designed a vibration auxiliary device with a two-stage lever amplification mechanism, achieving a maximum displacement of 75 μm and a frequency of 372.86 Hz, but the stiffness was weakened, and the step response was 85 ms. The maximum frequency during processing was 350 Hz, with a maximum displacement of 24 μm [29]. In summary, the performance of vibratory devices is limited by hinge size, mounting mode, and transmission chain dimensions. Optimizing these parameters can compromise performance, so selecting appropriate parameters based on practical scenarios is essential.

High-precision trajectory is essential for precision polishing, but structural design can cause discrepancies between actual and ideal motion. Improving device compliance reduces motion coupling. Dao et al. developed a lever amplification mechanism with minimal parasitic motion (0.02%) but a low natural frequency (112 Hz), limiting its suitability for vibration-assisted machining [30]. Zhang et al. designed a high-frequency (1000 Hz) vibration-cutting device with a 39 μm displacement and 6.5% coupling rate, but increased stiffness led to higher coupling errors [31]. Another design by Zhang et al. used a 2-DOF micro-displacement platform achieving 64.1 μm displacement at 1107.51 Hz with a 2.53% coupling error, simplifying the transmission chain for better performance [32]. The study by Yan et al. shows that from the analysis of the rotating magnetic pole center distance, the polishing force improved and the surface quality of the workpiece was improved [33]. Introducing rotary motion in non-resonant devices can enhance polishing by increasing the complexity of abrasive grain motion. Gu et al. developed a rotary vibration-assisted polishing system converting PZT linear motion to rotary motion, but axis offsets affected precision control [34]. To enhance vibration-assisted polishing, an RVAPD should convert PZT linear motion into rotary motion via a flexible hinge design, with optimized parameters and reduced stress for better performance.

Section 2 covers the design and working principle of the RVAPD. Section 3 develops a mathematical model for theoretical calculations. Section 4 uses finite element software to simulate performance and validate the design. Section 5 details offline physical testing, parameter validation, and guidance for vibration-assisted polishing. Section 6 presents the polishing experiment demonstrating RVAPD’s process improvement. The conclusion is in Section 7.

## 2. RVAPD Design and Working Principle

**Figure 1:**
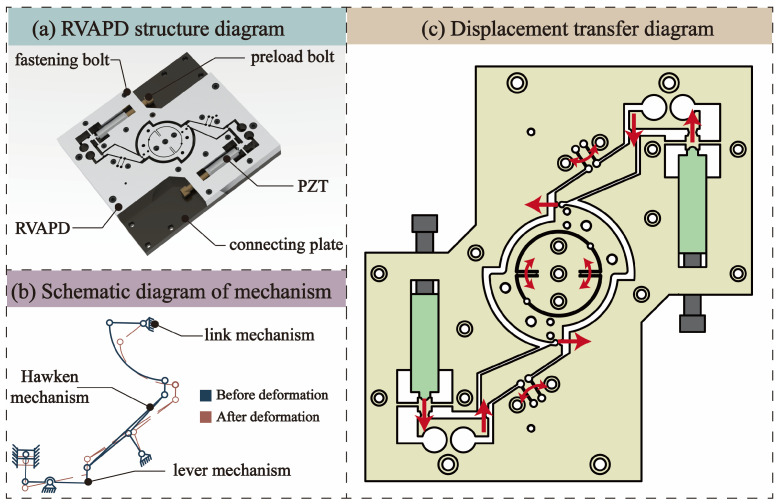
RVAPD structure composition and the movement principle. (**a**) RVAPD structure diagram. (**b**) Schematic diagram of mechanism. (**c**) Displacement transfer diagram.

The overall structure of the RVAPD includes the RVAPD unit, connecting plate, PZT, pre-tightening bolt, and tightening bolt. Figure 1a shows the RVAPD’s mechanical structure, where the PZT applies preload via a preload bolt for precise motion output. Two PZTs are arranged in parallel to provide the displacement and load for rotation around the Z-axis. The RVAPD’s design is compact to reduce mass and increase natural frequency, with a thick hinge design enhancing structural stiffness and vibration mode, and reducing maximum stress for long-term operation. The transmission mechanism consists of a rod and a Hoeckens mechanism, directly connecting the Hoeckens mechanism to the micro-moving platform. The crank performs circular motion, while the rocker terminal moves linearly. Figure 1b illustrates this. The size and shape of the lever mechanism play an important role in the transmission of displacement [35]. In this study, the rotation axis is set in the center of the lever; although this weakens the amplification of the lever, it can control the displacement of the PZT conversion direction, so that the size of the RVAPD and the structure are more compact. The link mechanism within the annular micro-platform ensures the platform returns to its original stress state. Arranged at 90°, the linkage and Hoeckens mechanisms stabilize RVAPD movement, improve stress distribution in the flexible hinge, and increase the load capacity of the micro-moving platform.

The PZT actuator simultaneously achieves displacement input and pushes the lever and the mass block. Using a Hoeckens mechanism, it promotes the tangential direction of crank movement, enabling linear movement along the tangential direction of the central platform at the end. Through the right circular hinges, the central platform transfers rotary torque. The symmetry of the mechanisms and the cooperation of the medial linkage allow for rotation around the Z-axis with only two displacement transfers. The displacement transfer of the RVAPD is illustrated in Figure 1c. Although there is some displacement loss, according to the design principle of the RVAPD, stiffness and compliance exhibit an inversely proportional relationship. However, increasing stiffness ensures transmission precision [36]. Based on the processing requirements, while satisfying the natural frequency of the RVAPD above 1000 Hz, the maximum stress value and the influence of location have been considered, and the output displacement and stiffness have been maximized.

## 3. RVAPD Static and Dynamic Model Analysis

To integrate the actual structure of the RVAPD with its mechanical basis and explore its key dimensions and performance indices, static and dynamic modeling must be performed. For static modeling and analysis of the RVAPD, common methods include the compliance matrix method [37], the pseudo-rigid body model method [38], and other techniques. Among these, the compliance matrix method, inspired by the finite element method, considers the flexible hinge as the flexible element and other parts as rigid components. This method offers high calculation accuracy and efficiency, making it suitable for modeling complex devices. For dynamics modeling, the Lagrangian method utilizes generalized forces and coordinates to derive the system’s dynamic equations without considering non-conservative forces, ensuring a simple calculation process. In this section, the input and output stiffness, as well as the natural frequency of the RVAPD, are theoretically analyzed using the compliance matrix method and the Lagrangian method.

### 3.1. Static Model Analysis

#### 3.1.1. Compliance Matrix Method

**Figure 2:**
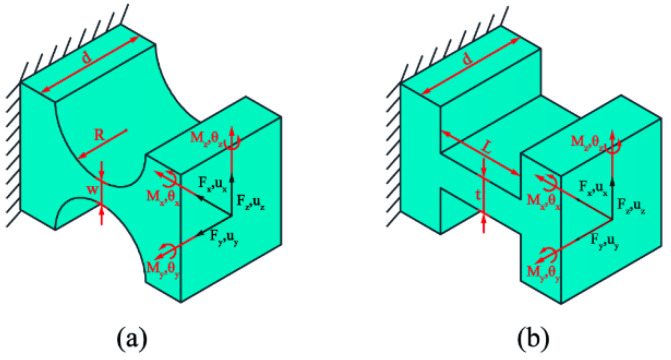
Key sizes of hinge, and the local coordinate system. (**a**) The local coordinate system of RCFHs. (**b**) The local coordinate system of LSFHs.

As a special motion pair, flexible hinges serve to convert material deformation into axial motion through angular displacement, offering high resolution, no mechanical friction, and machining integration. Flexible hinges exist in various forms. In the RVAPD, due to high displacement and accuracy requirements, leaf spring flexure hinges (LSFHs) and right circular flexure hinges (RCFHs) have been used as motion transmission mechanisms. The elastic model of the hinge has been based on spinor theory. When the load is applied to the end of the hinge, the flexible element produces slight deformation or movement. The deformation amount at the end of the flexible hinge can be represented by the motion spinor quantity U = ${\left(u_{x},\;u_{y},\;u_{z},\;\theta_{x},\;\theta_{y},\;\theta_{z}\right)}^{T}$, and the load spinor quantity W = ${\left(F_{x},\;F_{y},\;F_{z},\;M_{x},\;M_{y},\;M_{z}\right)}^{T}$ represents the load amount of the flexible hinge. The key dimensions of the hinges used and the local coordinate system are shown in Figure 2. The relationship between the deformation of the flexible hinge end and the load can be represented by Equation (1), where $C_{ij}$ is determined by the size of the flexible hinge itself:

(1) $$\left[\begin{array}{c} \begin{array}{c} u_{x}\\ u_{y}\\ u_{z} \end{array}\\ \begin{array}{c} \theta_{x}\\ \theta_{y}\\ \theta_{z} \end{array} \end{array}\right]=\left[\begin{array}{cccccc} C_{11} & 0 & 0 & 0 & 0 & 0\\ 0 & C_{22} & 0 & 0 & 0 & C_{15}\\ 0 & 0 & C_{33} & 0 & C_{24} & 0\\ 0 & 0 & 0 & C_{44} & 0 & 0\\ 0 & 0 & C_{42} & 0 & C_{55} & 0\\ 0 & C_{51} & 0 & 0 & 0 & C_{66} \end{array}\right]\left[\begin{array}{c} \begin{array}{c} F_{x}\\ F_{y}\\ F_{z} \end{array}\\ \begin{array}{c} M_{x}\\ M_{y}\\ M_{z} \end{array} \end{array}\right]$$

**Figure 3:**
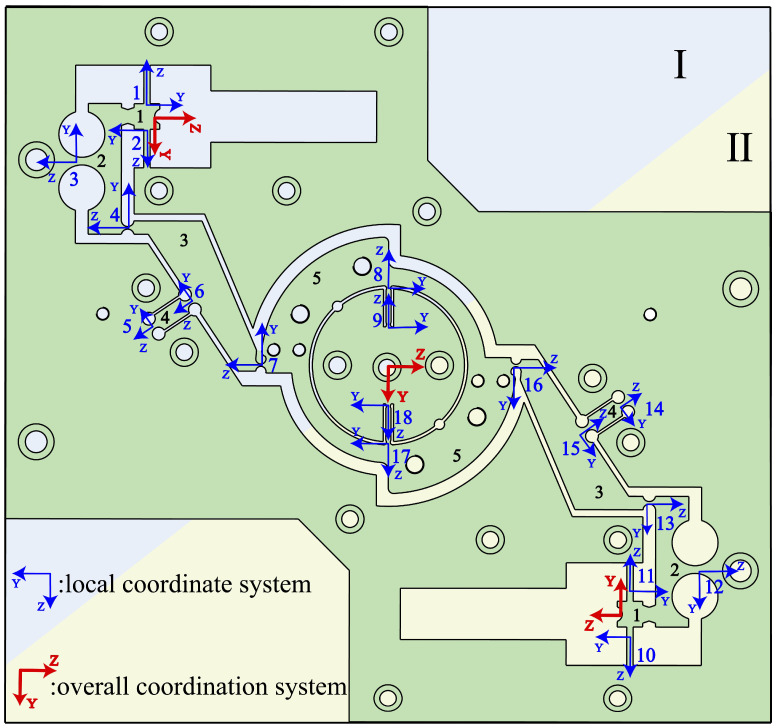
RVAPD local, global coordinate system.

Given that the RVAPD is centrosymmetric about the center point, it can be divided into two equal parts during the calculation process. Once the series–parallel relationship of the hinges in one of the parts has been established, the two parts can be connected in series in order to obtain the input and output stiffnesses of the entire device. As illustrated in Figure 3, the RVAPD can be partitioned into two distinct components. Given that the structural composition of Region I and Region II is identical, the input stiffness coordinate system will be represented in Region I, while the output stiffness coordinate system will be represented in Region II.

#### 3.1.2. Stiffness Modeling

In the motion characteristics of the device, stiffness is crucial for the selection of piezoelectric ceramics and the adjustment of the resonant frequency [39]. The input stiffness reflects the displacement of the input end of the RVAPD under different loads, while the output stiffness indicates the device’s ability to resist external disturbances. When calculating the input stiffness, Regions I and II are connected in parallel. The input compliance matrix $C_{in}$ of the RVAPD is expressed in Equation (2) according to the principle of adding parallel stiffnesses. Based on the principle that the compliance matrix and stiffness matrix are inverses, the output stiffness matrix $K_{in}$ of the RVAPD is expressed in Equation (3).

(2) $$C_{in}={\left[{\left(C_{I}^{O_{in}}\right)}^{-1}+{\left(C_{\mathrm{II}}^{O_{in}}\right)}^{-1}\right]}^{-1}$$

(3) $$K_{in}={\left(C_{in}\right)}^{-1}$$

(4) $$C_{I}^{O_{in}}={\left({\left(\sum_{i=1}^{2}C_{i}^{-1}+C_{3}^{-1}\right)}^{-1}+C_{4}+{\left(\sum_{i=5}^{6}C_{i}\right)}^{-1}\right)}^{-1}+\sum_{i=7}^{9}C_{i}$$

(5) $$C_{\mathrm{II}}^{O_{in}}={\left({\left(\sum_{i=10}^{11}C_{i}^{-1}+C_{12}^{-1}\right)}^{-1}+C_{13}+{\left(\sum_{i=14}^{15}C_{i}\right)}^{-1}\right)}^{-1}+\sum_{i=16}^{18}C_{i}$$

For Region I, the compliance and stiffness matrices of the hinge are summed according to the mode of action and distribution of the flexible hinge. The global coordinate system $O_{j}$ is defined at the input point of Region I. The input matrix $C_{in}^{I}$ of Region I is expressed in Equation (4), and the input matrix $C_{in}^{\mathrm{II}}$ of Region II is expressed in Equation (5). By substituting Equations (10) and (5) into Equation (2), the input stiffness of the RVAPD is found to be 51052 N/mm. To calculate the output stiffness, the global coordinate system $O_{j}$ is established at the center of the RVAPD, and the local coordinate system $C_{i}$ of each flexible hinge is rotated and translated relative to the global coordinate system $O_{j}$. Since the output stiffness and input stiffness calculations are based on the same flexible hinge frame, the force transmission relationship between the flexible hinges remains unchanged. Therefore, the series–parallel relationship between the flexible hinges remains unchanged in the output stiffness calculation. The output compliance matrix $C_{out}$ is represented by Equation (6). Since the compliance matrix and the stiffness matrix are inverses, the output stiffness matrix of the RVAPD is represented by Equation (7). The local coordinate system $C_{i}$ of each flexible hinge concerning the global coordinate system $O_{j}$ is transformed into Equations (8) and (9) by translation and rotation, and the output stiffness of the RVAPD is calculated to be 6145.8 N/mm.

(6) $$C_{out}={\left[{\left(C_{I}^{O_{out}}\right)}^{-1}+{\left(C_{\mathrm{II}}^{O_{out}}\right)}^{-1}\right]}^{-1}$$

(7) $$K_{out}={\left(C_{out}\right)}^{-1}$$

(8) $$C_{I}^{O_{out}}={\left({\left(\sum_{i=1}^{2}C_{i}^{-1}+C_{3}^{-1}\right)}^{-1}+C_{4}+{\left(\sum_{i=5}^{6}C_{i}\right)}^{-1}\right)}^{-1}+\sum_{i=7}^{9}C_{i}$$

(9) $$C_{\mathrm{II}}^{O_{out}}={\left({\left(\sum_{i=10}^{11}C_{i}^{-1}+C_{12}^{-1}\right)}^{-1}+C_{13}+{\left(\sum_{i=14}^{15}C_{i}\right)}^{-1}\right)}^{-1}+\sum_{i=16}^{18}C_{i}$$

### 3.2. Dynamic Modeling Analysis

The analysis of the natural frequency of the RVAPD constitutes an important part of the design calculation. The natural frequency of the RVAPD has been calculated using the Lagrangian method, which offers high calculation accuracy. To simplify and unify the dynamic performance analysis, the mass–spring model has been used to describe the dynamic performance of the RVAPD. The equivalent mass and moment of inertia of the moving parts, such as the central platform and the connecting rod, have been applied to the central position of the central platform. The rotational stiffness has been applied to the rotational pair of each flexible hinge. The input force is applied at the driving point of each piezoceramic drive shaft, and the contact stiffness is considered in series with the equivalent stiffness. The Lagrange equations are presented in Equation (10). Here, $E_{k}$ represents the kinetic energy of the system, *q* is the displacement variable, $E_{p}$ is the potential energy of the system, and $F_{i}$ is the generalized force. The RVAPD kinetic energy is the sum of the kinetic energies of each mass, as represented in Equation (17).

(10) $$\frac{d}{dt}\left(\frac{\partial E_{k}}{\partial \dot{q}}\right)-\frac{\partial E_{k}}{\partial q}+\frac{\partial E_{p}}{\partial q}=F_{i}$$

(11) $$E_{k}=\frac{1}{2}M_{i}v_{i}^{2}+\frac{1}{2}J_{i}w_{i}^{2}$$

$M_{i}$ represents the mass of the i-th component, $J_{j}$ represents the moment of inertia, and $w_{j}$ represents the angular velocity. The total kinetic energy $E_{k}$ of the RVAPD is obtained by substituting the parameters of all parts of the RVAPD into Equation (11), as shown in Equation (12). Here, $\dot{y_{i}}$ is the derivative of the displacement of each mass with respect to time, $L_{i}$ is the moment arm length, and $\dot{w_{i}}$ is the derivative of the angular velocity with respect to time. The relationship between the equivalent mass $m_{i}$ of the RVAPD and the total kinetic energy $E_{k}$ is given by Equation (13), and the equivalent stiffness $K_{i}$ is derived from Equation (14). The natural frequency f of the RVAPD is determined from Equation (15), and the first-order frequency of the RVAPD has been calculated to be 1305.08 Hz.

(12) $$E_{k}=2\times \left(\begin{array}{c} \frac{1}{2}m_{1}{\left(\dot{y_{in}}\right)}^{2}+\frac{1}{2}m_{2}{\left(\dot{y_{1}}\right)}^{2}+\frac{1}{2}\left(\frac{1}{12}\cdot m_{2}\cdot {L_{2}}^{2}\right){\left(\dot{w_{2}}\right)}^{2}+\frac{1}{2}m_{3}{\left(\dot{y_{2}}\right)}^{2}\\ +\frac{1}{2}\left(\frac{1}{12}\cdot m_{3}\cdot {L_{3}}^{2}\right){\left(\dot{w_{3}}\right)}^{2}+\frac{1}{2}m_{4}{\left(\dot{y_{3}}\right)}^{2} \end{array}\right)+\frac{1}{2}m_{5}{\left({\dot{y}}_{out}\right)}^{2}$$

(13) $$E_{k}=\frac{1}{2}m_{i}{\left({\dot{y}}_{out}\right)}^{2}$$

(14) $$K_{i}=\frac{F_{in}}{y_{out}}$$

(15) $$f=\frac{1}{2\pi }\sqrt{\frac{K_{i}}{m_{i}}}$$

## 4. Finite Element Simulation of RVAPD

To further investigate the dynamic and static performance of the RVAPD, finite element analysis was performed using ABAQUS software (version 2022). 7075Al-T6 was chosen as the material for RVAPD due to its advantages of high rigidity, low density, and low cost [40]. The natural frequency, input and output stiffness, maximum stress, and maximum displacement of the RVAPD were obtained.

### 4.1. Modal Analysis

**Figure 4:**
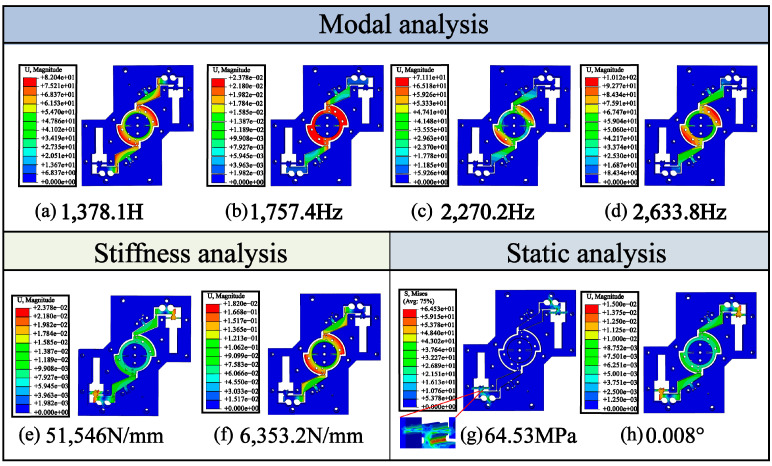
RVAPD finite element simulation. (**a**) First-order frequency. (**b**) Second-order frequency. (**c**) Third-order frequency. (**d**) Fourth-order frequency. (**e**) Input stiffness. (**f**) Output stiffness. (**g**) Maximum stress. (**h**) Maximum displacement.

High dynamic response capability is essential for vibration-assisted machining, and modal analysis has been an effective method for evaluating mechanical structure and system dynamic performance. ABAQUS finite element analysis software was utilized to analyze the RVAPD and the mode cloud diagrams of the first four orders of the vibration platform were obtained, as shown in Figure 4a–d. The natural frequencies of the first four orders were determined as 1378.1 Hz, 1757.4 Hz, 2270.2 Hz, and 2633.8 Hz, respectively. Compared with the theoretical first-order natural frequency, the error is only 5.298%. The first-order modal vibration mode of deformation is consistent with the actual working output deformation. Therefore, during machining, it was necessary to keep the device deformation within a certain range, typically under the first-order frequency condition. The second and higher frequencies differed significantly from the first frequency, and the corresponding mode shapes did not affect the movement at frequencies below the first-order frequency.

### 4.2. Static Analysis

#### 4.2.1. Stiffness of the Finite Element Mode

Equal and opposite forces of 1000 N, 1200 N, 1500 N, 1800 N, and 2000 N were applied to the left and right ends of the RVAPD, respectively, resulting in displacements of 19.4 µm, 23.2 µm, 29.1 µm, 34.9 µm, and 38.9 µm at the input end, as shown in Figure 4e. From the simulation data, the input stiffness of the non-resonant vibration platform was calculated to be 51,546 N/mm, which is significantly lower than the stiffness of the PZT and meets the design requirements. Compared to the theoretical calculation, the error is only 0.958%.

Using the same method, a force of 1000 N was applied to both ends of the center platform simultaneously, and the resulting output stiffness was determined to be 6353.2 N/mm, as shown in Figure 4f. Compared to the calculated results, the error is 3.2%. The results of the numerical comparison between theoretical calculations and finite element simulations are presented in Table 1.

#### 4.2.2. Maximum Stress Analysis

In the practical application process of RVAPD, to ensure the sustainability of RVAPD processing, the maximum stress should remain below the allowable stress of material use; otherwise, RVAPD could be damaged. The maximum stress position of the vibration platform was obtained through stress analysis conducted using ABAQUS software. The limit stress of 7075Al-T6 is 524 MPa, with a safety coefficient of s = 1.8; thus, the allowable stress of the material was calculated by Equation (16).

(16) $$\left[\sigma \right]=\frac{\sigma_{s}}{s}=291.11\;\mathrm{Mpa}$$

As shown in Figure 4g, when an external force of 1000 N is applied to the two input ends of the RVAPD, the maximum stress $\sigma_{max}$ of the RVAPD is 64.53 MPa, which is significantly below the allowable stress of the material. The maximum stress was found to occur at the weak point of the LSFH. This indicates that the RVAPD consistently operates within the deformation range of linear elasticity and is capable of performing long-term tasks with high repeatability.

#### 4.2.3. The Maximal Displacement Analysis

Since the RVAPD can be considered a spring-damping system, the external force generated increases with the displacement of the piezoelectric ceramic. Part of the displacement of the piezoelectric ceramic resists the external force, and the loss is influenced by the input stiffness $K_{in}$ of the RVAPD. The maximum displacement *L* of the piezoelectric ceramic under different load stiffness is obtained from Equation (17) [41]. Here, $L_{0}$ represents the maximum displacement of the piezoelectric ceramic without load, $K_{T}$ is the stiffness of the piezoelectric ceramic itself, and $K_{F}$ is the load stiffness, which in this context is the input stiffness of the RVAPD. The maximum displacement of the piezoelectric ceramic at this input stiffness $K_{in}$ was calculated to be 12.41 µm. By applying this input displacement to the input ends on both sides, the maximum input displacement is 0.008°, as shown in Figure 4h.

(17) $$L=L_{0}*\frac{K_{T}}{K_{T}+K_{F}}$$

## 5. RVAPD Performance Test

### 5.1. RVAPD Physical Prototype

**Figure 5:**
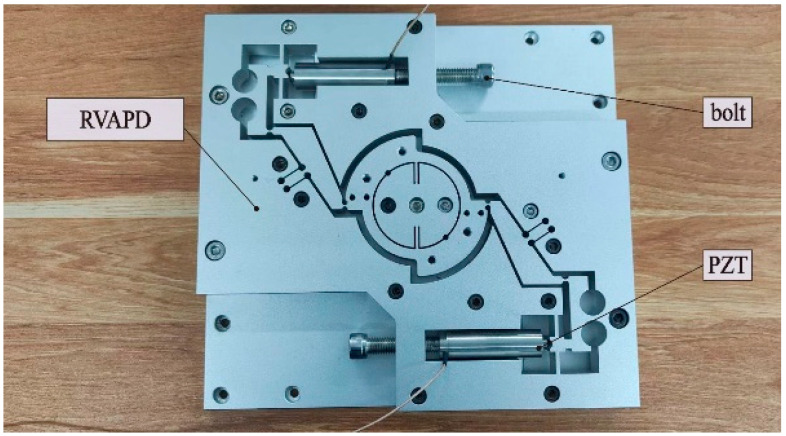
RVAPD physical prototype.

A prototype of the RVAPD was fabricated using wire EDM, as shown in Figure 5. The material selected was 7075Al-T6, which possesses a Young’s modulus of 71,700 MPa and a density of 2810 kg/m^3^. The PZT (PSt150V/10/60VS20, Harbin Core Tomorrow Science & Technology Co., Ltd. (Harbin, China)) was placed in the slot and pre-tightened with bolts. The maximum displacement of the PZT under the maximum voltage (150 Vpp) was 60 µm without load. The overall dimensions of the vibration auxiliary device were 300 mm × 280 mm × 22 mm.

### 5.2. Work Frequency and Workspace Test

#### 5.2.1. Test Platform Construction

**Figure 6:**
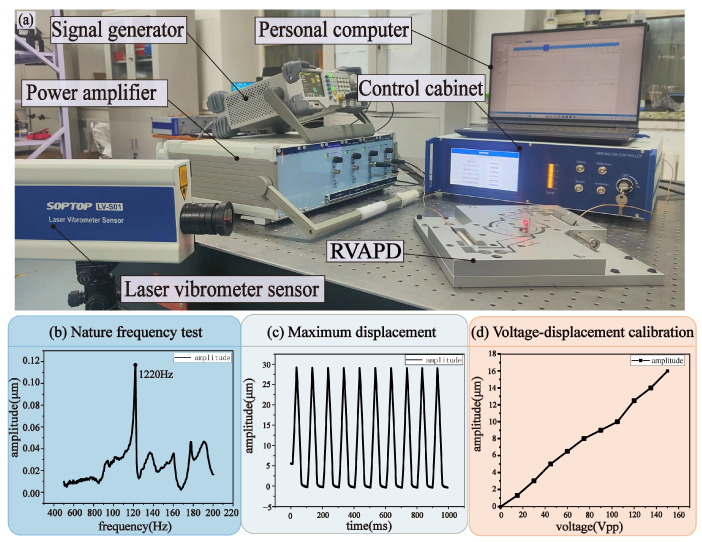
The test platform construction and test results. (**a**) The test setup diagram. (**b**) Nature frequency. (**c**) Maximum displacement. (**d**) Voltage–displacement calibration.

To verify the working frequency range and operational space of the RVAPD, the natural frequency and maximum displacement of the RVAPD are tested in this section, and the corresponding tests at different voltages and RVAPD displacement were carried out offline. The test setup diagram is shown in Figure 6a. The test was performed on an air float isolation platform. In the offline test, the signal generator (DG1062Z, Rigol Technologies Co., LTD. (Suzhou, China)) was used to output two identical signals. Through the power amplifier (E00.C4, Harbin Core Tomorrow Science & Technology Co., Ltd. (Harbin, China)), it was delivered to the input end of the PZT excitation vibration auxiliary device on both sides to drive the rotation of the center platform. Since the output displacement is small, the output signal was recorded as a straight-line displacement of a moving contour point along its tangent direction. Using a laser vibrometer sensor (LV-S01, Sunny Optical Intelligence Technology Co., LTD. (Hangzhou, China)), the output displacement of the RVAPD was observed. The observed signals were transmitted to a personal computer through the control cabinet and recorded.

#### 5.2.2. Working Frequency Test

To verify the operating frequency range of the RVAPD, the side wall of the central platform of the vibration auxiliary device was struck with a force hammer to force it to vibrate freely, and the displacement response was analyzed by a laser vibrometer sensor. The test results are shown in Figure 6b. The natural frequency of the RVAPD was measured as 1220 Hz, which is lower than the value of the finite element simulation. The assembly mode of RVAPD and the accumulation of manufacturing errors caused differences between the analysis model, finite element simulation, and actual test. The experimental results show that the RVAPD meets the wide operating frequency range required by the experiment.

#### 5.2.3. Workspace Test

The RVAPD exhibited the largest displacement, reflecting its characteristics in terms of the largest movement amplitude, as driven by the PZT test results as shown in Figure 6c. Tests with a 10 Hz, 10 Vpp parameter, on both sides using the triangle wave signal simultaneously motivated the PZT to produce its maximum value. The maximum displacement of the vibrator was measured at 29 μm. The discrepancy between the simulation and the actual test results can be attributed to the fact that the load used in the finite element simulation was a constant load, which remains unaltered by external influences. However, the effect produced by the PZT in the actual test was influenced by the load stiffness, fixation effect, and manufacturing error, resulting in an input force that exceeded the rated output force of the PZT and an output displacement that was higher than the simulation value.

The vibration amplitude in the machining process directly affects the machining outcome. To utilize the vibration auxiliary device for vibration-assisted processing, it is necessary to record the displacement caused by different voltages. A sinusoidal signal was used for driving because of its unique advantages in signal analysis and processing. The voltage amplitude of the input signal was varied by the signal generator, and the 50 Hz sinusoidal signal was used to stimulate the vibration auxiliary device and record the output displacement. The results are shown in Figure 6d. The displacement of the vibrator was observed to increase linearly with the increase in voltage. It has been demonstrated that the vibration auxiliary device can provide variable and stable vibration signals for vibration-assisted processing and enable parameter selection in the processing process.

### 5.3. Output Stiffness Test

#### 5.3.1. Test Platform Construction

**Figure 7:**
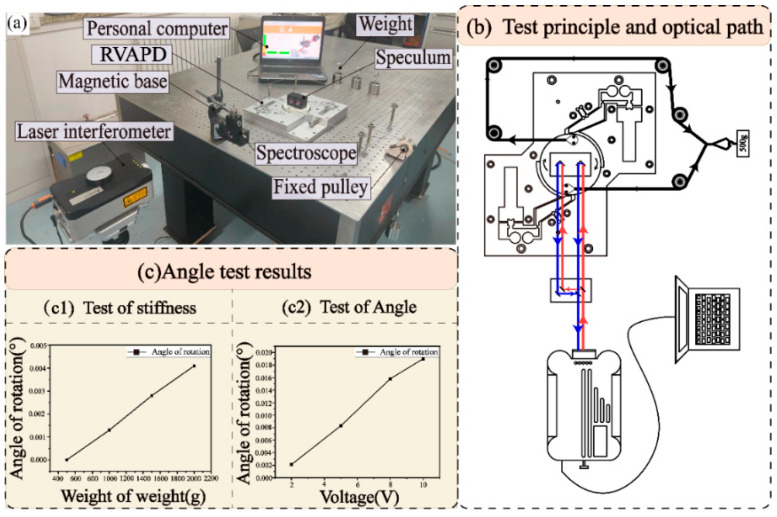
RVAPD local, global coordinate system. (**a**) The test setup diagram. (**b**) Test principle and optical path. (**c**) Angle test results.

To test and verify the movement of the vibration assist device and its advantages in resistance factors, an output stiffness test platform was set up, as shown in Figure 7a. A rated and steadily increasing force was applied to the vibratory drive by a 500 g weight. The nylon rope and fixed pulley transmitted the force to both ends of the central platform of the vibrator, causing the vibrator to make a rotational movement. A laser interferometer (XL-80, Renishaw PLC) issued a laser beam that returned after passing through a spectroscope and speculum interferometer, illustrating the output stiffness testing principle and the optical path structures, as shown in Figure 7b. The rotational movement of the central platform was recorded by the interferometer by changing the optical path difference in the laser beam via the mirror.

#### 5.3.2. Output Stiffness Test

The test results are shown in Figure 7(c1). The initial value on the personal computer was set to 0°, and the reading increased to 0.0013°, 0.0028°, and 0.0041° after three weights were hung sequentially. By dividing the weight by the displacement, the output stiffness test value was calculated as 4454 N/mm. The reasons why the measured result is lower than the simulation include the following: (1) the whole device being bolted together, and the tightness of the bolts seriously affecting the rigidity value; (2) the driving force being transmitted by the nylon rope and the bolt, with the bolt being forced in the upper part, and the middle and lower parts driving the platform. In this process, the bolt was not perpendicular to the air-floating platform, and the movement displacement of the platform was smaller than the movement distance of the weight, so the measured stiffness was lower than the simulated stiffness. The test results showed that the designed anti-vibration device exhibited high output rigidity, ensuring the stability of the movement during vibration.

#### 5.3.3. Maximum Angle Test

The test results are shown in Figure 7(c2), where 2 V, 5 V, 8 V, and 10 V voltages were applied to the RVAPD, and the rotation angle was measured by a laser interferometer. With the increase in the excitation voltage, the rotation angles were measured as 0.002°, 0.008°, 0.016°, and 0.019°, respectively, proving that the designed RVAPD has a large angle control space.

### 5.4. Static Performance Test

#### 5.4.1. Test Platform Construction

**Figure 8:**
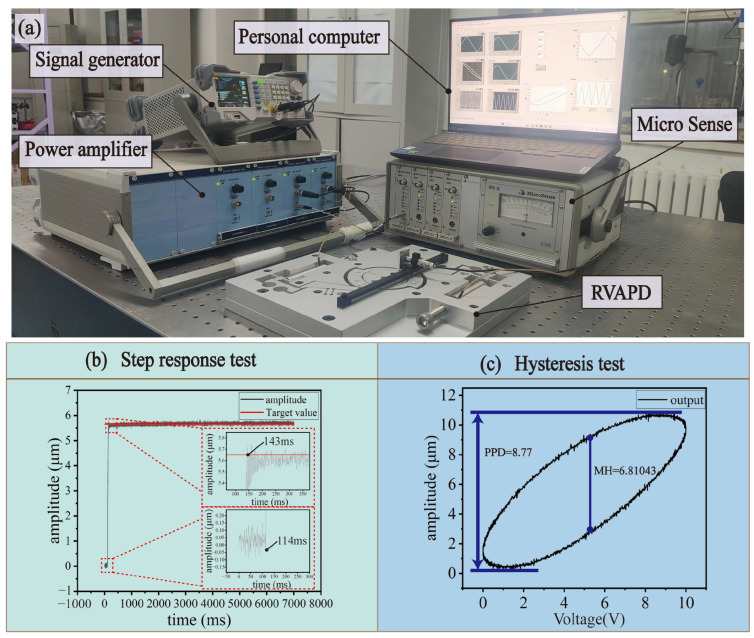
Static performance test platform setup and test results. (**a**) The test setup diagram. (**b**) Step response test. (**c**) Hysteresis test.

Low-frequency signals were used to excite the PZT to reflect the static performance of the vibrator. In this section, Micro Sense was used to collect the displacement signal of the central platform to test the step response and hysteresis effect of the RVAPD. The static performance test setup is shown in Figure 8a.

#### 5.4.2. Step Response Test

To verify the localization capability of the RVAPD, the response time was tested with the target displacement set to 5.5 μm, as shown in Figure 8b. The total measurement time was 7 s, the initial response time was 114 ms, the time to reach the target displacement was 143 ms, and the response time was 29 ms. Although there were clear stabilization errors and overshoot, the RVAPD still accurately executed the external commands with a fast response time, suitable for the vibration-assisted polishing process.

#### 5.4.3. Hysteresis Test

Hysteresis is an inherent phenomenon in PZTs and this phenomenon will degrade the performance and positioning accuracy of RVAPD [42], so it is necessary to test for hysteresis. The hysteresis effect can be effectively mitigated by a flexible structural design. As shown in Figure 8c, at 0.5 Hz and 5 μm driving signal, the peak-to-peak displacement (PPD) of the RVAPD was measured at 8.77 μm, and the maximum hysteresis (MH) was measured at 6.81043 μm, indicating its suitability for the vibration-assisted polishing process.

## 6. Vibration-Assisted Polishing Experiment

To demonstrate the polishing performance of the RVAPD, vibration-assisted and non-vibration-assisted machining experiments were conducted on SiC workpieces. The improvement effect of the RVAPD on the polishing results was verified by measuring the change in force during the polishing process and the final surface quality.

### 6.1. Experimental Platform Construction

**Figure 9:**
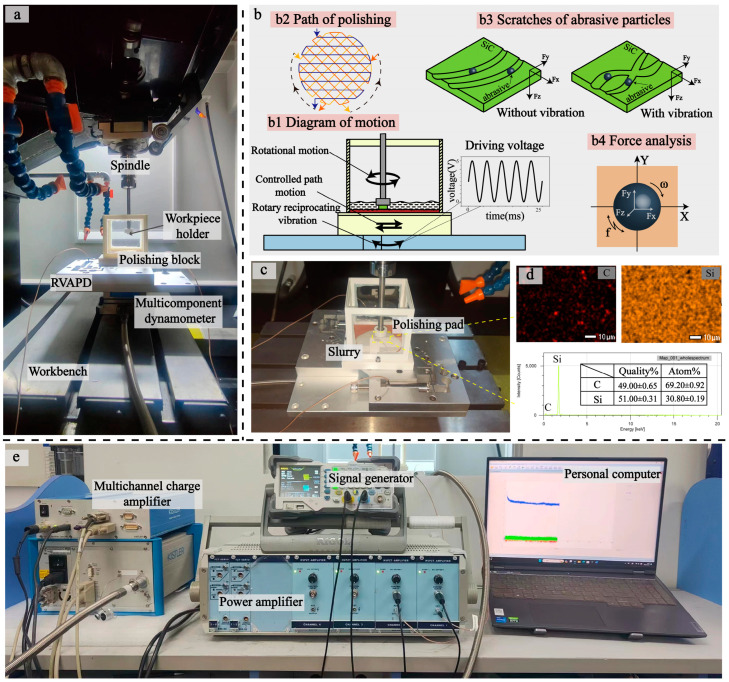
Vibration-assisted polishing experiment building and polishing principle. (**a**) Diagram of the experimental platform. (**b**) Schematic diagram of the experiment. (**c**) Diagram of polishing area. (**d**) EDS images of SiC. (**e**) Signal processing equipment.

**Figure 10:**
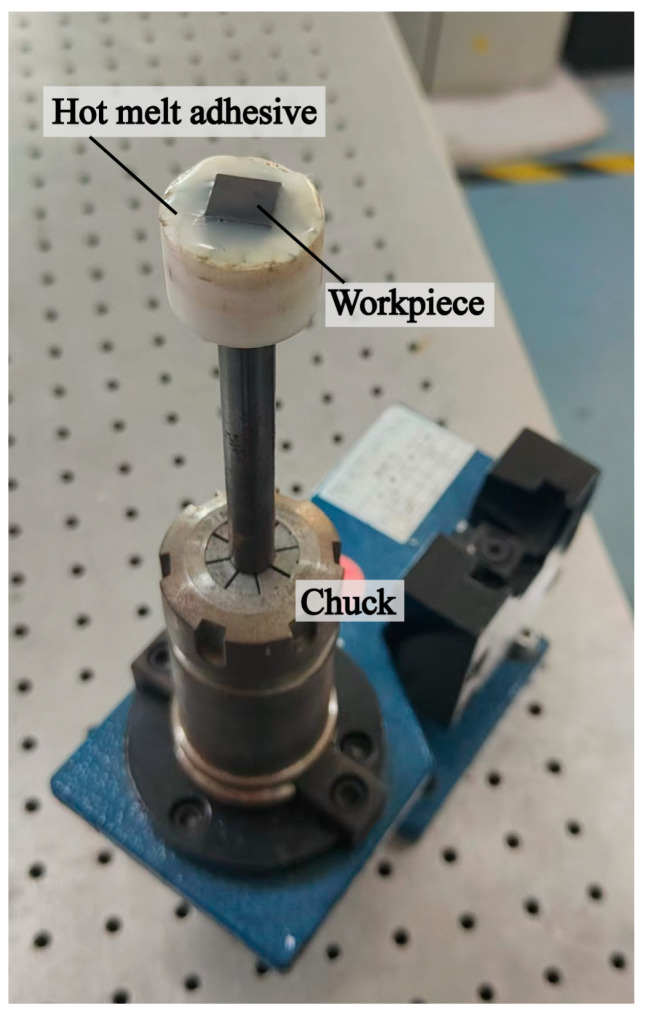
Detail of workpiece chuck.

As shown in Figure 9a, the vibration-assisted machining experiment was carried out on a commercial machine tool (KEMT-MT52). The RVAPD was bolted to the multi-component dynamometer, and the multi-component dynamometer was bolted to the T-slots of the workbench. It was controlled by the motion path of the workbench. The 3D-printed polishing block formed the polishing area, and the polishing pad was attached to the bottom of the polishing block with backside adhesive, immersed in slurry from Xinhui Technology Co., Ltd. (Shenzhen, China), as shown in Figure 9c. SiC artifacts were fixed with hot melt adhesive to a fixture made by 3D printing. The detail diagram of the workpiece chuck is shown in Figure 10, and the dimension of the workpiece is 10 mm × 10 mm. The SiC EDS images are shown in Figure 9d. The fixtures were connected by adhesive to the spindle for high-speed rotary motion, and the vibration-assisted machining motion is illustrated in Figure 9(b1). To more fully participate in the polishing process, the polishing pad followed the path shown in Figure 9(b2). Measured force signals were accepted and displayed by a multi-channel charge amplifier on the personal computer, as shown in Figure 9e. The force analysis of single diamond grit in the vibration field is shown in Figure 9(b4). The impact of vibration on grinding grains resulted in overlapping independent scraping paths, as shown in Figure 9(b3), increasing the complexity of the abrasive particle trajectory and improving the efficiency of material removal.

### 6.2. The Polishing Force Experiment

**Figure 11:**
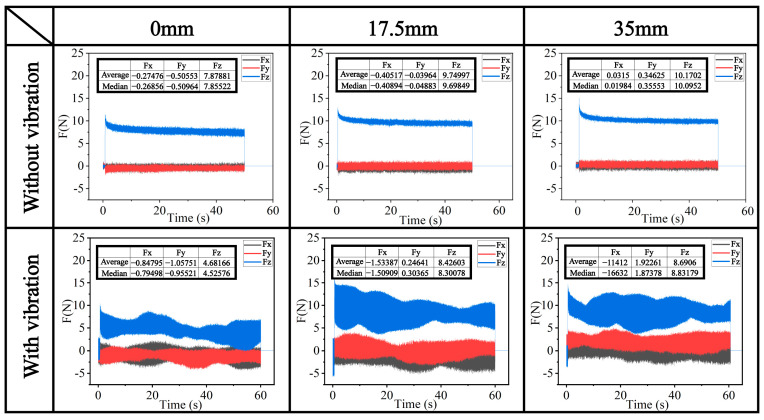
Polishing force test results.

To verify the effect of the rotating polishing pad on the polishing force, fixed-point polishing experiments were conducted at three positions inside the polishing tank: 0 mm, 17.5 mm, and 35 mm from the center. The results are shown in Figure 11. The sliding abrasive has better removal capability and reduces sub-surface damage compared to rolling abrasive motion [43]. A consistent downward pressure of 50 μm was applied as pre-pressure during polishing. Without vibration, the polishing force in all three directions remains almost constant. However, with a vibration parameter of 200 Hz, and 8 μm, the polishing force shows a periodic trend corresponding to the vibration frequency: the tangential force increases, while the normal force decreases. The variation in polishing force slightly increases with distance from the center. The changes at 17.5 mm and 35 mm are similar due to the comparable polishing speeds at these locations.

### 6.3. Surface Quality

**Figure 12:**
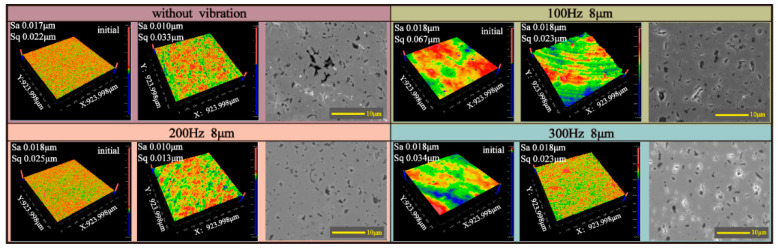
SiC ceramic surface roughness measurements.

To verify the improvement effect of RVAPD on the polishing process of SiC ceramics, vibration-assisted and non-vibration-assisted polishing experiments were conducted. The combination of low rotational and scanning speeds enables the effective removal of material while simultaneously reducing the wear of the polishing pad [44]. Following experimentation to ascertain the impact of rotational and scanning speeds on roughness, the rotational speed was determined to be 1000 rpm, while the scanning speed was set at 100 mm/min, and the polishing time was 30 min. And the processed surfaces were measured by a white-light interferometer and a scanning electron microscope. The surface roughness of the workpieces was 18 nm ± 1 nm, as shown in Figure 12. The results showed that the average height deviation (Sa) of the surface roughness of the workpieces was reduced from 18 nm to 10 nm without vibration polishing; at the optimum vibration parameter (200 Hz), the Sa was also reduced to 10 nm, indicating that the polishing process had an improvement effect on the surface of the workpieces. However, compared with the mean height deviation of surface roughness (Sa), the root square deviation of surface roughness (Sq) showed a large difference. Sq was reduced from 25 nm to 13 nm after the introduction of vibration, and Sq did not show a significant trend of reduction or even increase without vibration-assisted polishing. The introduction of vibration reduced the degree of dispersion of the surface roughness of the material and had a positive effect on the improvement in the homogeneity of the surface of the workpiece. In the SEM image, it can be observed that the cratering and fragmentation of the material surface have been reduced and improved under the optimal vibration parameters.

## 7. Conclusions

A novel rotary vibration-assisted polishing device (RVAPD) is proposed, in which the linear motion of the piezoelectric transducer (PZT) is converted into rotary motion of the central platform by means of a flexible mechanism. The objective of the device is to meet the requirements of vibratory polishing through the reasonable layout of each mechanism, with the additional design objectives of a high frequency, a compact structure, and low stress. The theoretical analysis of the RVAPD is conducted using the flexibility matrix method and Lagrange equations, while the simulation analysis is performed with the ABAQUS software. The discrepancy between the theoretical and simulated results is less than 10%. The results of the experimental tests indicate that the RVAPD has an intrinsic frequency of 1220 Hz, a maximum rotation angle of up to 0.019°, and an output stiffness of 4454 N/mm. These characteristics enable the device to provide vibration-assisted polishing with a wide range of adjustable parameters and a high degree of vibration stability. Vibration-assisted polishing experiments on SiC ceramics were conducted using the designed RVAPD. The polishing tangential force exhibited an increasing trend following the application of vibration. Under the optimal vibration parameters, the Sa of surface roughness was reduced to 10 nm, and Sq was reduced to 13 nm, resulting in enhanced surface uniformity. The presence of surface cratering and fragmentation was observed in SEM.

## Figures and Tables

**Table 1:** The theory and simulation results.

	First-Order Frequency	Input Stiffness	Output Stiffness
Model analysis	1305.08 Hz	51,052 N/mm	6145.8 N/mm
Simulation analysis	1378.10 Hz	51,546 N/mm	6353.2 N/mm
Error	5.298%	0.958%	3.2%

## Data Availability

The original contributions presented in this study are included in the article; further inquiries can be directed to the corresponding authors.
